# Spermatozoa lacking Fertilization Influencing Membrane Protein (FIMP) fail to fuse with oocytes in mice

**DOI:** 10.1073/pnas.1917060117

**Published:** 2020-04-15

**Authors:** Yoshitaka Fujihara, Yonggang Lu, Taichi Noda, Asami Oji, Tamara Larasati, Kanako Kojima-Kita, Zhifeng Yu, Ryan M. Matzuk, Martin M. Matzuk, Masahito Ikawa

**Affiliations:** ^a^Research Institute for Microbial Diseases, Osaka University, Suita, 565-0871 Osaka, Japan;; ^b^Center for Drug Discovery and Department of Pathology & Immunology, Baylor College of Medicine, Houston, TX 77030;; ^c^Department of Bioscience and Genetics, National Cerebral and Cardiovascular Center, Suita, 564-8565 Osaka, Japan;; ^d^Laboratory for Developmental Epigenetics, RIKEN Center for Biosystems Dynamics Research, Kobe, 650-0047 Hyogo, Japan;; ^e^Graduate School of Medicine, Osaka University, Suita, 565-0871 Osaka, Japan;; ^f^The Institute of Medical Science, The University of Tokyo, Minato-ku, 108-8639 Tokyo, Japan

**Keywords:** CRISPR/Cas9, fertilization, infertility, IZUMO1, transgenic

## Abstract

Sperm–oocyte fusion is a critical event in mammalian fertilization, categorized by three indispensable proteins. Sperm membrane protein IZUMO1 and its counterpart oocyte membrane protein JUNO make a protein complex allowing sperm to interact with the oocyte, and subsequent sperm–oocyte fusion. Oocyte tetraspanin protein CD9 also contributes to sperm–oocyte fusion. However, the fusion process cannot be explained solely by these three essential factors. In this study, we focused on analyzing a testis-specific gene *4930451I11Rik* and generated mutant mice using the CRISPR/Cas9 system. Although IZUMO1 remained in *4930451I11Rik* knockout (KO) spermatozoa, the KO spermatozoa were unable to fuse with oocytes and the KO males were severely subfertile. 4930451I11Rik encodes two isoforms: a transmembrane (TM) form and a secreted form. Both CRISPR/Cas9-mediated TM deletion and transgenic (Tg) rescue with the TM form revealed that only the TM form plays a critical role in sperm–oocyte fusion. Thus, we renamed this TM form Fertilization Influencing Membrane Protein (FIMP). The mCherry-tagged FIMP TM form was localized to the sperm equatorial segment where the sperm–oocyte fusion event occurs. Thus, FIMP is a sperm-specific transmembrane protein that is necessary for the sperm–oocyte fusion process.

## Significance

As the human body is composed of 60 trillion cells that originate from a fertilized egg, sperm–oocyte fusion is the initial event of our life. Few sperm–oocyte fusion factors have been unveiled to date, and only IZUMO1 has been identified as a sperm-specific fusion-mediating protein. Here, we identified the testis-specific *4930451I11Rik* gene important for male fertility, playing a role in sperm–oocyte fusion during fertilization. Based on its functional role, we renamed this gene fertilization influencing membrane protein (*Fimp*). We discovered a factor responsible for sperm–oocyte fusion in mammals, and this knowledge could be used to develop in vitro and in vivo infertility treatments as well as male contraceptives.

Mammalian fertilization comprises various steps including sperm migration through the female reproductive tract, changes in sperm physiology and morphology, sperm–egg interaction, and fusion (1, 2). Recent gene KO mouse studies revealed essential factors for fertilization (3–5). However, to date, only three proteins have been shown to play an essential role in sperm–oocyte fusion, with all three expressed on the cell surface. CD9 and JUNO modulate female fertility, while IZUMO1 regulates fusion in spermatozoa. CD9 consists of a group of tetraspanin membrane proteins and is expressed ubiquitously. However, while *Cd9* KO females appeared healthy, their fertility was significantly reduced (6–8). *Cd9* KO oocytes show impaired development of microvilli on the oocyte membrane and are unable to fuse with spermatozoa (9). IZUMO1 is a type I transmembrane protein localized on the sperm acrosomal membrane. One of the physiological changes of spermatozoa during the acrosome reaction is the presentation of IZUMO1 onto sperm surface. IZUMO1 translocates from the acrosomal membrane to the plasma membrane and spreads over the equatorial and postacrosomal regions. As such, IZUMO1 is used as a marker of the acrosome reaction (10, 11). *Izumo1* KO male mice are sterile because spermatozoa lack the ability to fuse with the oocyte plasma membrane (12). Although the cytoplasmic tail of IZUMO1 is dispensable for fertility, the N-terminal region (5th through 113th amino acids) is found to be essential for sperm–oocyte fusion (13, 14). GPI-anchored protein JUNO (officially named IZUMO1R) was identified as the oocyte receptor of IZUMO1, and *Juno* KO female mice were also infertile due to impaired sperm–oocyte fusion (15). Further structural analyses found that several amino acid residues of each protein are critical for the direct binding of JUNO and IZUMO1 in mice and humans (16–20). However, IZUMO1–JUNO interaction is not enough to trigger membrane fusion (21). Therefore, additional factors must contribute to the molecular mechanisms of sperm–oocyte fusion.

KO mouse studies have identified many essential genes for fertility by conventional gene-targeting approaches (1, 5). However, the ES cell-mediated gene targeting and subsequent chimeric mice production are laborious and costly. In 2013, the CRISPR/Cas9-mediated genome editing system emerged, enabling large-scale screening using KO mice (22, 23). In fact, while we have reported 96 reproductive tract enriched genes that are individually dispensable (24–27), we have also reported 18 genes and 2 gene clusters that are essential for male fertility (12, 28–43) CRISPR/Cas9 technologies have allowed us not only to disrupt a gene but also to analyze important regions of a gene of interest. One can easily delete protein domains or introduce a point mutation at the nucleotide level (31, 44).

Here, we focused on analyzing a testis-specific gene *4930451I11Rik* that is localized on mouse chromosome 7 and that is conserved in humans and other mammals. To examine the physiological role of this gene, we generated CRISPR/Cas9-mediated KO mice. The *4930451I11Rik* KO male mice were severely subfertile as a result of impaired sperm–oocyte fusion, even though IZUMO1 remained intact. Moreover, we found that 4930451I11Rik is expressed as two isoforms (transmembrane [TM] and secreted forms) in the testis due to alternative splicing. We generated 4930451I11Rik TM-deleted mice using the CRISPR/Cas9 system. The 4930451I11Rik TM-deleted mice demonstrated that the TM form is critical for spermatozoa to fuse with oocytes. Thus, we renamed 4930451I11Rik as fertilization influencing membrane protein (FIMP). In addition, the TM form of *Fimp-mCherry* Tg mice restored male infertility in *Fimp* KO mice and was detected on the sperm equatorial segment where the sperm–oocyte fusion event occurs. Thus, we have discovered FIMP as the second sperm factor that is necessary for spermatozoa to fuse with oocytes.

## Results

### Testis-Specific Expression of *4930451I11Rik* in Mice and Humans.

**Fig. 1.**
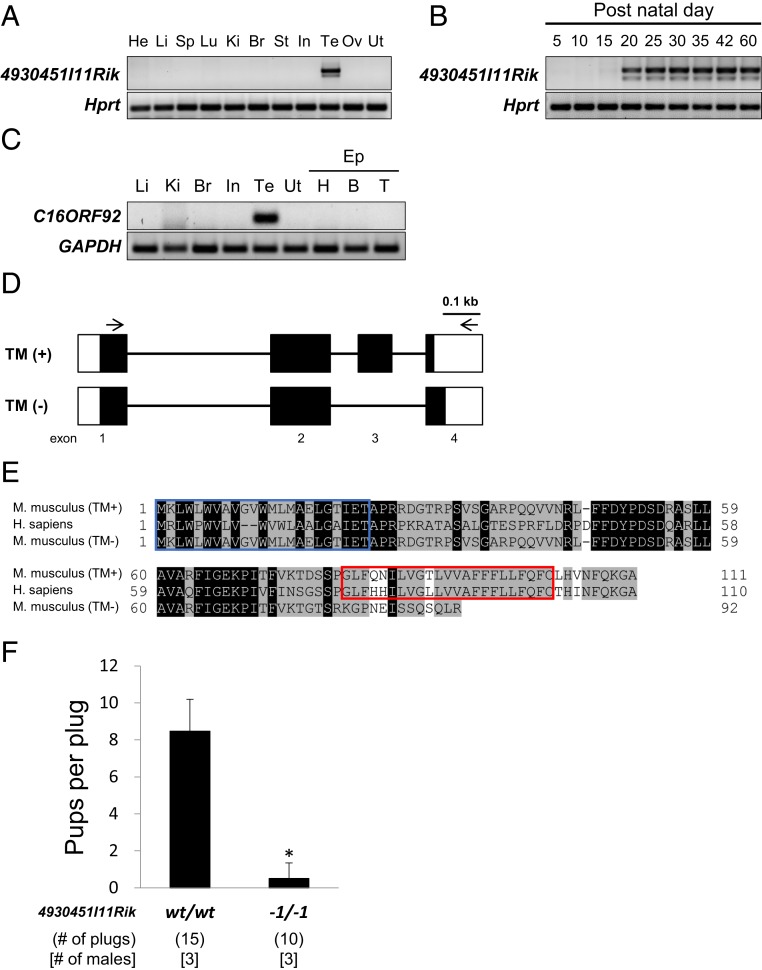
Characterization of *4930451I11Rik* in mice and humans and male fertility in *4930451I11Rik* KO mice. (*A*) Testis-specific expression of mouse *4930451I11Rik* by multitissue RT-PCR analysis. The expression of each gene was examined by RT-PCR, using RNA isolated from various organs. *4930451I11Rik* was detected only in the mouse testis. The *Hprt* gene was used as an expression control. He, heart; Li, liver; Sp, spleen; Lu, lung; Ki, kidney; Br, brain; St, stomach; In, intestine; Te, testis; Ov, ovary; Ut, uterus. (*B*) RT-PCR analysis of mouse *4930451I11Rik* in mouse testis. *4930451I11Rik* was expressed 20 d after birth. (*C*) RT-PCR analysis of human *C16ORF92* (ortholog of mouse *4930451I11Rik*) in human tissues. Human *C16ORF92* was also detected only in the testis. The *GAPDH* gene was used as an expression control. Ep, epididymis; H, head (caput epididymis); B, body (corpus epididymis); T, tail (cauda epididymis). (*D*) Two isoforms, TM form and secreted form, of mouse *4930451I11Rik* gene. The *4930451I11Rik* mRNA consists of four exons. Although the TM (+) form contains all four exons, the TM (−) form expresses only three exons, as the third exon is spliced out. Exons are indicated by boxes. Protein-coding and noncoding regions are indicated in black and white, respectively. The sequences of the two isoforms are shown in *SI Appendix*, Fig. S1*A*. Arrows indicate primers used for RT-PCR analysis. (*E*) Amino acid sequence similarity of mouse 4930451I11Rik and human C16ORF92. The length of TM (+) form and TM (−) form of the mouse 4930451I11Rik protein is 111 and 92 amino acids, respectively. The amino acids 1 to 77 are identical between TM (+) and (−) forms. Human C16ORF92 has 110 amino acids, and 71 amino acids match between mice (TM [+] form) and humans. The signal peptide region and putative transmembrane domain are indicated by the blue and red boxes, respectively. Black indicates a match in all sequences, whereas gray indicates a match in two sequences. (*F*) Average litter size of *4930451I11Rik* KO males. The average litter size was measured by the number of pups in each copulatory plug. The mean (± SD) litter size was 8.5 ± 1.7 in females mated with wild-type (wt) males and 0.5 ± 0.8 in females mated with *4930451I11Rik* KO (−1/−1) males. *P < 0.01, Student’s *t* test.

Mouse *4930451I11Rik* gene is localized to chromosome 7. The expression of this gene in various mouse organs was examined by RT-PCR, and it showed testis-specific expression (Fig. 1*A*). Next, the onset of *4930451I11Rik* expression in testes was examined by RT-PCR. This gene was expressed 20 d after birth (Fig. 1*B*). Furthermore, the human ortholog *C16ORF92* showed testis-specific expression as well (Fig. 1*C*). Since we detected two bands (312 bp and 400 bp) of *4930451I11Rik* from the mouse testes (Fig. 1 *A* and *B*), we confirmed their identities using direct sequencing analysis (*SI Appendix*, Fig. S1*A*). The *4930451I11Rik* consists of four exons and is expressed in two variants from three or four exons, respectively. The difference between these two variants is whether the third exon encoding the transmembrane domain is present or not (Fig. 1*D*). We found that mouse *4930451I11Rik* has two isoforms, TM (+) and secreted (TM [−]; Fig. 1*E*). Protein structure analysis predicted that the TM form of 4930451I11Rik is a type I single-pass transmembrane protein containing a cleavable N-terminal signal peptide and a transmembrane-encoding sequence (*SI Appendix*, Fig. S1 *B* and *C*). Moreover, the protein sequence of 4930451I11Rik is highly conserved in mammals; the gene has been observed in more than 70 eutherian mammals including marsupials (wallaby; *SI Appendix*, Fig. S1*D*; http://www.informatics.jax.org/homology/52331). These data indicate that mouse *4930451I11Rik* and human ortholog *C16ORF92* are both testis-enriched genes. While mouse 4930451I11Rik is expressed in two forms, TM and secreted, human C16ORF92 is only expressed as the TM form.

### Male Fertility of *4930451I11Rik* KO Mice.

To analyze the physiological role of 4930451I11Rik, we generated *4930451I11Rik* KO mice by CRISPR/Cas9. A 1-bp deletion in the second exon of *4930451I11Rik* was confirmed by PCR and direct sequencing analysis (*SI Appendix*, Fig. S2*A*). The 1-bp deletion caused a frameshift mutation that resulted in a premature termination codon at the 43rd amino acid of 4930451I11Rik (TM [+]: 111 amino acids and TM [−]: 92 amino acids in wild-type mice; *SI Appendix*, Fig. S2*B*). Thus, both isoforms were disrupted by the 1-bp deletion. The KO (−1/−1) mice had no overt developmental abnormalities. Furthermore, no deleterious effects on testicular histology and sperm morphology were observed (*SI Appendix*, Fig. S2 *C* and *D*). To examine male fertility, adult KO males were mated with wild-type females for several months. The KO males were severely subfertile, showing normal mating behavior with successful ejaculation and vaginal plug formation. The average number of pups per plug was 8.5 ± 1.7 in wild-type males (15 plugs) and 0.5 ± 0.8 in the KO males (10 plugs; Fig. 1*F*). These data indicated that *4930451I11Rik* is required for normal male fertility in mice.

### In Vitro Fertilizing Ability in *4930451I11Rik* KO Spermatozoa.

**Fig. 2.**
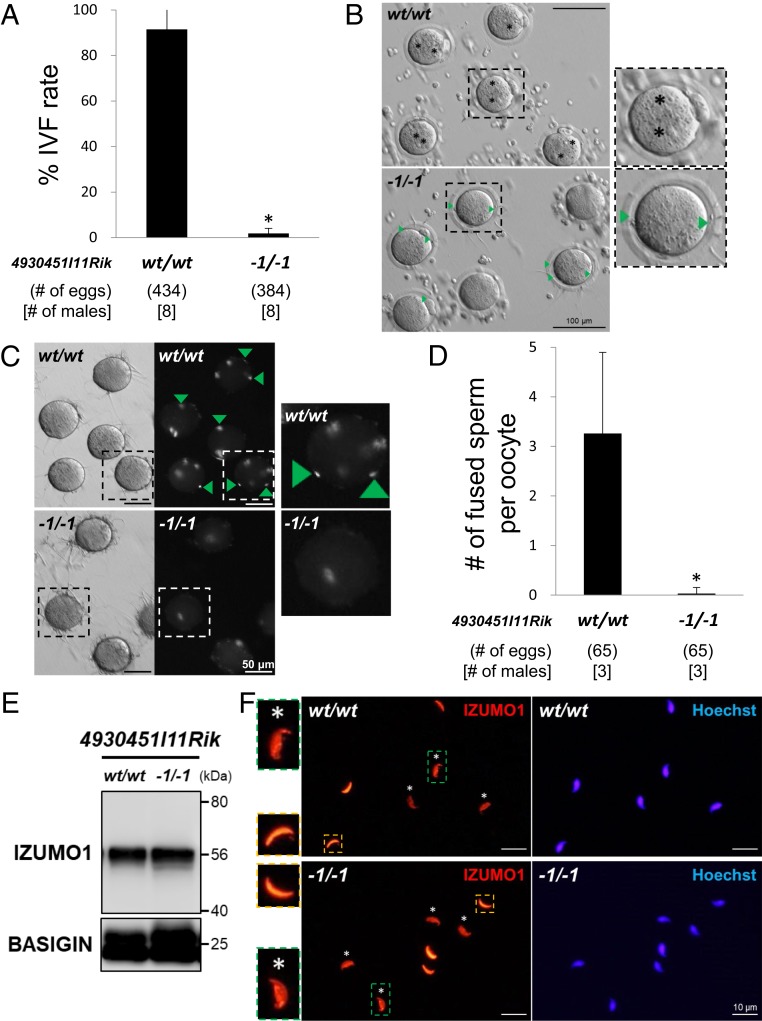
In vitro fertilizing ability of *4930451I11Rik* KO spermatozoa. (*A*) IVF rates using wild-type and *4930451I11Rik* KO spermatozoa. Average fertilization rates of wild-type (wt) and *4930451I11Rik* KO (−1/−1) spermatozoa were 91.5 ± 9.3% (397/434 eggs) and 1.8 ± 2.2% (7/384 eggs), respectively. *P < 0.01, Student’s *t* test. (*B*) Observation of spermatozoa and oocytes 7 h after insemination. Although oocytes inseminated with wild-type (wt) spermatozoa formed pronuclei (indicated by asterisks), oocytes inseminated with *4930451I11Rik* KO (−1/−1) spermatozoa formed no visible pronuclei. Rather, spermatozoa were localized to the perivitelline space. *4930451I11Rik* KO spermatozoa could not properly fuse with the oocyte membrane. Pronuclei are indicated by asterisks. Green arrowheads indicate spermatozoa that remain in the perivitelline space after penetrating the zona pellucida. Enlarged images are indicated by dotted squares. (Scale bars: 100 μm.) (*C*) Observation of sperm–oocyte fusion in wild-type and *4930451I11Rik* KO spermatozoa. *4930451I11Rik* KO spermatozoa have impaired oocyte membrane fusion ability in vitro. Fused spermatozoa are indicated by green arrowheads. Enlarged images are indicated by dotted squares. (Scale bars: 50 μm.) (*D*) Average number of spermatozoa fused with the oocyte membrane in vitro. The number of fused spermatozoa in *4930451I11Rik* KO (−1/−1) mice (0.03 ± 0.1 spermatozoa; mean ± SD) was significantly reduced compared with that of wild-type (wt) mice (3.3 ± 1.6 spermatozoa). *P < 0.01, Student’s *t* test. (*E*) Immunoblot analysis of IZUMO1 using sperm lysates collected from wild-type and *4930451I11Rik* KO mice. IZUMO1 is a sperm membrane protein essential for sperm–oocyte fusion. Although *4930451I11Rik* KO (−1/−1) spermatozoa showed impaired ability to fuse with the oocyte membrane, IZUMO1 was still present in *4930451I11Rik* KO spermatozoa. BASIGIN was used as a loading control. (*F*) Immunostaining of IZUMO1 in *4930451I11Rik* KO spermatozoa. IZUMO1 is used as a marker of the acrosome reaction. The acrosome reaction occurred in *4930451I11Rik* KO (−1/−1) spermatozoa. Acrosome reaction is determined by IZUMO1 localization (acrosome-intact spermatozoa: acrosomal cap pattern [orange dotted squares], acrosome-reacted spermatozoa: entire head pattern [green dotted squares]). Acrosome-reacted spermatozoa are indicated by asterisks. Enlarged images are indicated by dotted squares. (Scale bars: 10 μm.)

To analyze the cause of infertility in *4930451I11Rik* KO males, we performed in vitro fertilization (IVF) assays using cauda epididymal spermatozoa from *4930451I11Rik* KO males with cumulus-intact oocytes. *4930451I11Rik* KO spermatozoa could penetrate the zona pellucida (ZP), but rarely fertilize oocytes; 91.5 ± 9.3% (397/434) fertilized eggs were observed when oocytes were treated with wild-type spermatozoa, whereas 1.8 ± 2.2% (7/384) fertilized eggs were observed with KO spermatozoa (Fig. 2*A*). While we were able to observe the pronuclei of control eggs 7 h after insemination, *4930451I11Rik* KO spermatozoa remained in the perivitelline space (Fig. 2*B*). Next, we measured the fusion ability of the spermatozoa mixed with ZP-free oocytes in vitro. *4930451I11Rik* KO spermatozoa had a significantly reduced ability to fuse with the oocyte membrane compared with wild-type (3.3 ± 1.6 and 0.03 ± 0.1 spermatozoa per egg using wild-type and KO spermatozoa, respectively; *P* < 0.01; Fig. 2 *C* and *D*). The KO spermatozoa showed impaired ability to fuse with the oocytes.

To examine the effects of 4930451I11Rik disruption on IZUMO1, we performed an immunoblot analysis of IZUMO1 in *4930451I11Rik* KO spermatozoa. There were no significant differences in the size or amount of IZUMO1 between wild-type and *4930451I11Rik* KO spermatozoa (Fig. 2*E*). Next, we observed IZUMO1 localization by immunostaining analysis. IZUMO1 changes its localization from the acrosomal cap to the entire head during the acrosome reaction, which is considered one of the most essential steps for fertilization. When we observed the cauda epididymal spermatozoa 4 h after incubation, there was no difference in the IZUMO1 staining pattern in *4930451I11Rik* KO spermatozoa and in wild-type (Fig. 2*F*). These results indicate that IZUMO1 remains and correctly translocates in *4930451I11Rik* KO spermatozoa.

### Analysis of 4930451I11Rik TM-Deleted Mice.

**Fig. 3.**
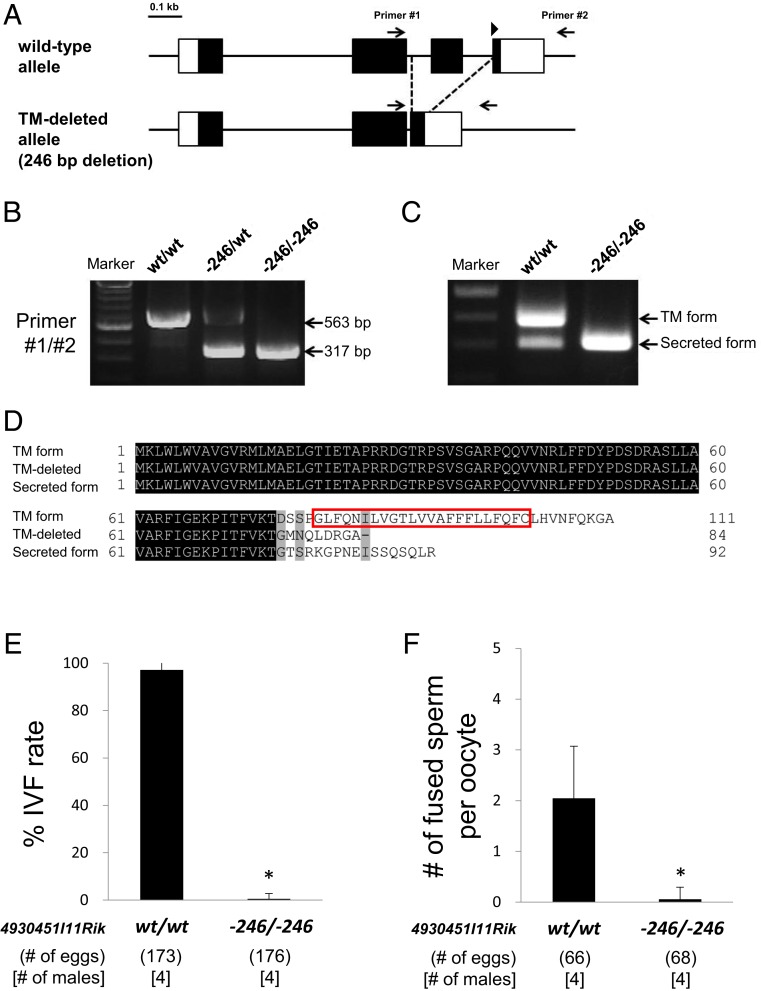
Analysis of 4930451I11Rik TM-deleted mice. (*A*) Targeting scheme of the 246-bp deletion in the *4930451I11Rik* locus. The third exon that encodes TM domain of 4930451I11Rik was deleted by the CRISPR/Cas9 system. Arrowhead indicates gRNA targeting in the fourth exon. (Scale bar: 0.1 kb.) (*B*) Genotyping using PCR in 4930451I11Rik TM-deleted mice. Both a 563-bp band representing the wild-type (wt/wt) allele and a 317-bp band representing the TM-deleted (−246/−246) allele were amplified by PCR using primers #1 and #2. (*C*) RT-PCR analysis of 4930451I11Rik TM-deleted mouse testis. The coding region of *4930451I11Rik* was amplified by PCR in the testis from TM-deleted mice. The TM band (upper band in wild-type testis) disappeared from 4930451I11Rik TM-deleted (−246/−246) mouse testis as expected. The lower band consisted of the secreted form and the in-frame mutation due to the 246-bp deletion as confirmed by direct sequencing (*SI Appendix*, Fig. S3*B*). (*D*) Amino acid sequence alignments of 4930451I11Rik from wild-type and 4930451I11Rik TM-deleted mice. A total of 90.5% of amino acids (76/84 amino acids) are identical with the secreted form of 4930451I11Rik. Black indicates a match in all sequences, whereas gray indicates a match in two sequences. (*E*) IVF rates using wild-type and 4930451I11Rik TM-deleted spermatozoa. Average fertilization rates of wild-type (wt) and 4930451I11Rik TM-deleted (−246/−246) spermatozoa were 97.1 ± 2.5% (168/173 eggs) and 0.6 ± 1.3% (1/176 eggs), respectively. *P < 0.01, Student’s *t* test. (*F*) Average number of fused spermatozoa with oocyte membrane in vitro. The number of fused spermatozoa in 4930451I11Rik TM-deleted (−246/−246) mice (0.1 ± 0.2 spermatozoa; mean ± SD) was significantly reduced compared with that of wild-type (wt) mice (2.0 ± 1.0 spermatozoa). *P < 0.01, Student’s *t* test.

Our data indicate that the 4930451I11Rik is an important factor for the sperm–oocyte fusion process. As shown in Fig. 1, 4930451I11Rik is expressed as two isoforms in the mouse testis: the TM form and the secreted form. Thus, to determine which isoform is essential, we deleted the third exon that encodes a TM region by the CRISPR/Cas9 system (Fig. 3*A*). A 246-bp deletion around the third exon of *4930451I11Rik* was confirmed by PCR, direct sequencing (Fig. 3*B* and *SI Appendix*, Fig. S3*A*), and RT-PCR analysis (Fig. 3*C* and *SI Appendix*, Fig. S3*B*). The 246-bp deletion (including 88 bp of the third exon and 12 bp of fourth exon) caused an in-frame mutation that appeared from a premature terminal codon at the 85th amino acid of 4930451I11Rik (Fig. 3*D*). We verified that only the TM form of 4930451I11Rik disappeared from the testis in 4930451I11Rik TM-deleted mice (Fig. 3*C*). We performed IVF assays to check the fertilizing ability of 4930451I11Rik TM-deleted mouse spermatozoa. Similar to the KO (−1/−1) mice in which both secreted and TM forms were disrupted, 4930451I11Rik TM-deleted spermatozoa could rarely fertilize oocytes (0.6 ± 1.3%; 1 fertilized egg detected out of 176 oocytes/embryos analyzed) in contrast to wild-type spermatozoa, in which nearly all oocytes were fertilized (97.1 ± 2.5%; 168 eggs/173 oocytes/embryos analyzed; Fig. 3*E*). 4930451I11Rik TM-deleted spermatozoa also had a significantly reduced ability to fuse with the oocyte membrane compared with the control (2.0 ± 1.0 and 0.1 ± 0.2 spermatozoa per egg in the wild-type and TM-deleted spermatozoa, respectively; *P* < 0.01; Fig. 3*F*). Moreover, IZUMO1 was present and localized properly in the TM-deleted mice, as well as wild-type mice (*SI Appendix*, Fig. S3 *C* and *D*). To signify this newly discovered sperm–oocyte fusion-mediating factor, we renamed 4930451I11Rik to fertilization influencing membrane protein (FIMP). Thus, the TM form of FIMP is important for the sperm–oocyte fusion process and functions independent of IZUMO1 in spermatozoa.

### Analysis of *Fimp-mCherry* Tg Rescue Mice.

**Fig. 4.**
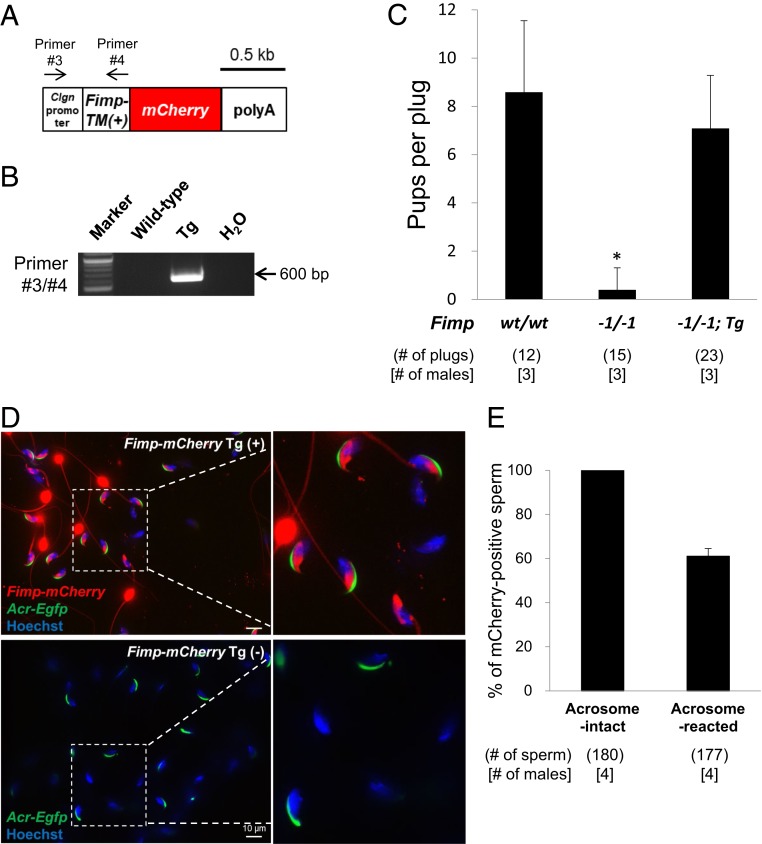
Analysis of *Fimp-mCherry* Tg rescue mice. (*A*) Design of *Fimp-mCherry* transgene. Transgene designed in which the transmembrane isoform of FIMP and mCherry are expressed as a fused protein under the testicular germ cell-specific *Clgn* promoter (total, 1.9 kb). Arrows indicate primers used for genotyping. (Scale bar: 0.5 kb.) (*B*) Genotyping with PCR in *Fimp-mCherry* Tg mice. A 600-bp band representing the Tg allele was amplified by PCR using primers #3 and #4. (*C*) Average litter size of *Fimp-mCherry* Tg rescued *Fimp* KO male mice. The *Fimp-mCherry* transgene restored male infertility in *Fimp* KO mice. The average litter size was measured by the number of pups in each copulatory plug. The mean (± SD) litter size was 8.6 ± 3.0 in wild-type (wt) males, 0.4 ± 0.9 in *Fimp* KO (−1/−1) males, and 7.1 ± 2.2 in *Fimp-mCherry* Tg rescued *Fimp* KO (−1/−1; Tg) male mice. *P < 0.01, Student’s *t* test. (*D*) Confocal microscopic observation of *Fimp-mCherry* Tg mouse spermatozoa. The TM form of FIMP and mCherry fused protein (red signals) were localized to the equatorial segment of the sperm head in acrosome-intact spermatozoa (Acr-EGFP [green signals] positive). However, after the acrosome reaction (Acr-EGFP [green signals] negative), both fluorescent and nonfluorescent spermatozoa were observed. Red signals could not be detected in *Acr-Egfp* Tg mouse spermatozoa (without *Fimp-mCherry* transgene). (Scale bars: 10 μm.) (*E*) Fate of FIMP-mCherry protein on spermatozoa after the acrosome reaction. Although all of the acrosome-intact spermatozoa express FIMP-mCherry, 39.0 ± 3.5% of acrosome-reacted spermatozoa lacked FIMP-mCherry in the sperm head (mean ± SD). Acrosome-reacted spermatozoa were observed after 3 h incubation.

To observe the localization of FIMP, we generated *Fimp-mCherry* Tg mice because no anti-FIMP antibodies are available for immunostaining and immunoblot analyses. The transgene expressed the mCherry-tagged TM form of FIMP (111 aa) under the testicular germ cell-specific *Clgn* promoter (Fig. 4*A*). To confirm that the *Fimp-mCherry* transgene is functional, we obtained Tg mice on a *Fimp* KO (−1/−1) background (Fig. 4*B*). The infertile phenotype of *Fimp* KO males was fully rescued by the transgene (8.6 ± 3.0, 0.4 ± 0.9, and 7.1 ± 2.2 pups per plug in the wild-type [12 plugs], *Fimp* KO [15 plugs], and Tg rescued *Fimp* KO [23 plugs] males, respectively; *P* < 0.01; Fig. 4*C*). These results confirm that the TM form of FIMP regulates the sperm–oocyte fusion process. Next, we observed testicular sections and cauda epididymal spermatozoa in *Fimp-mCherry* Tg mice. Although spermatogenesis looked normal in testicular sections, the mCherry protein accumulated in the lumen of seminiferous tubules (*SI Appendix*, Fig. S4*A*). Because most of the mCherry protein was concentrated in a cytoplasmic droplet on the sperm tail, it was difficult to check the localization of FIMP-mCherry on the sperm head by conventional fluorescence microscopic observation (*SI Appendix*, Fig. S4*B*). By confocal microscopic observation, we confirmed that FIMP-mCherry was present on all sperm heads, specifically on the equatorial segment, before the acrosome reaction (*SI Appendix*, Fig. S4*C*). However, both FIMP-mCherry fluorescent and nonfluorescent (39.0 ± 3.5%) acrosome-reacted spermatozoa were observed (Fig. 4 *D* and *E*). These data indicated that the TM form of the FIMP protein is localized to the equatorial segment of the sperm head and can restore the infertility of *Fimp* KO males.

### In Vitro Cell–Oocyte Binding Assay.

Cultured cells transiently expressing mouse IZUMO1 can tightly bind to the plasma membrane of ZP-free mouse oocytes (14). To confirm the relationship of FIMP and the oocyte membrane in sperm–oocyte interactions, we performed cell–oocyte binding assays using COS-7 and HEK293T cells expressing IZUMO1 and FIMP (*SI Appendix*, Figs. S4 *D* and *E* and S5). As shown in *SI Appendix*, Figs. S4*E* and S5*B*, FIMP-expressing cells could not bind to the oocyte surface, whereas IZUMO1-expressing cells could (4.0 ± 0.4 cells per oocyte in IZUMO1-expressing cells). Moreover, coexpression of IZUMO1 and FIMP in transfected HEK293T cells also did not enhance the binding ability to the oocyte membrane (4.5 ± 0.5 and 4.1 ± 0.1 cells per oocyte in IZUMO1-expressing cells and IZUMO1 and FIMP coexpressing cells, respectively; *SI Appendix*, Fig. S5*C*). Thus, these results suggest that FIMP does not bind directly to the oocyte surface in sperm–oocyte fusion and does not directly promote IZUMO1 binding to JUNO on the oocyte plasma membrane.

## Discussion

Before the advances in gene manipulation technology, oocyte and sperm proteins important for fertilization were discovered via IVF experiments using biochemical approaches. However, in the last 2 decades, KO strategies have revealed that many “key” sperm proteins uncovered in vitro using antibody blocking or peptide competition experiments are not essential for in vivo fertilization (1, 5). In 2005, we found that the sperm membrane protein IZUMO1 is essential for spermatozoa to fuse with oocytes using the KO approach (12). Almost 10 years later, GPI-anchored protein JUNO, the oocyte receptor of IZUMO1, was reported by Bianchi et al. (15). They identified JUNO by an avidity-based extracellular interaction screen (AVEXIS) assay that combines polymerized IZUMO1 and an iterative expression cloning approach using a mouse egg cDNA library. Recently, crystal structure analysis of the IZUMO1 and JUNO complex provided data on the amino acids critical in forming the intercellular bridge necessary for initiating sperm–oocyte fusion (45).

In this article, we focused on a testis-enriched gene *4930451I11Rik* and generated genetically modified mice that have a single gene disruption by the CRISPR/Cas9 system. We found that *4930451I11Rik* is necessary for the fertilizing ability of spermatozoa (Figs. 1*F* and 2*A*). Although *4930451I11Rik* KO mice produced normal-appearing epididymal spermatozoa, they showed impaired sperm–oocyte fusion (Fig. 2*B* and *SI Appendix*, Fig. S2*D*). Unexpectedly, although IZUMO1 is still intact, *4930451I11Rik* KO spermatozoa lacked the ability to fuse with oocytes (Fig. 2*E*). Thus, using CRISPR/Cas9, we have uncovered a KO mouse that produces sperm that cannot fuse with oocytes even though IZUMO1 remains. IZUMO1 changes its localization on the sperm head (from the acrosomal cap to the entire head) during the acrosome reaction, one of the most critical steps of fertilization (10, 11). This IZUMO1 translocation step is required for the ability of spermatozoa to fertilize eggs as evaluated by *Spesp1* (sperm equatorial segment protein 1) and *Tssk6* (testis specific serine kinase 6) KO mice, which both showed reduced sperm fusion ability (46, 47). Since SPESP1 and TSSK6 are localized to the sperm head, abnormal sperm head formation may cause impaired translocation of IZUMO1 in these KO spermatozoa. However, IZUMO1 translocation was normal in *4930451I11Rik* KO spermatozoa (Fig. 2*F*). Our results indicate that 4930451I11Rik is a transmembrane protein that regulates the sperm–oocyte fusion process via an IZUMO1-unrelated step.

Based on these results, we renamed 4930451I11Rik to fertilization influencing membrane protein (FIMP). *Fimp* is expressed as two variants in the mouse testis (Fig. 1 *A* and *B*). Direct sequencing and RT-PCR analysis confirmed the two forms: either localized to the transmembrane or secreted (Fig. 1*D* and *SI Appendix*, Fig. S1 *A*–*C*). In this study, we took advantage of the CRISPR/Cas9 system for quick and efficient genetic modification (48–51) of the *Fimp* allele in mice. To determine which isoform is essential for male fertility, we generated FIMP TM-deleted mice which introduced an in-frame mutation in the TM form (91.3% similarity of the secreted form; Fig. 3*D*). The TM-deleted mice showed the same phenotypes of *Fimp* KO mice in which both forms were disrupted (Fig. 3 *E* and *F* and *SI Appendix*, Fig. S3 *C* and *D*). Protein sequence alignment also shows that there is no secreted form among other mammalian species including humans (*SI Appendix*, Fig. S1*D*). Moreover, a transgene expressing the FIMP TM form restored male infertility in *Fimp* KO mice (Fig. 4*C*). Tg mouse analysis showed that FIMP is localized to the sperm equatorial segment membrane, which is attached to the oocyte membrane for fusion (*SI Appendix*, Fig. S4*C*). However, there were ∼40% of acrosome-reacted spermatozoa in which the FIMP-mCherry signals disappeared, in contrast to acrosome-intact spermatozoa, where their signals were localized to the equatorial segment (Fig. 4 *D* and *E*). The in vitro cell-oocyte binding assay showed that FIMP-expressing cells do not bind to the oocyte plasma membrane, in direct contrast to IZUMO1-expressing cells that directly bind to the oocyte surface (*SI Appendix*, Figs. S4*E* and S5*B*). Further studies are required to determine which population of acrosome-reacted spermatozoa (those that still express FIMP and/or those that do not) are required for the sperm–oocyte fusion process and how the TM form of FIMP (111 aa) regulates sperm fertilization. Although FIMP-expressing cells do not bind the oocyte in vitro, our results indicate that the TM form of FIMP is localized to the sperm equatorial segment to function during fusion with oocytes. FIMP may play a role after the initiation of the IZUMO1–JUNO complex for sperm–oocyte membrane binding, and/or FIMP could be part of a larger IZUMO1-independent sperm complex required for sperm–oocyte interactions.

In conclusion, our *Fimp* KO mice may prove useful in elucidating the physiological function of human FIMP (C16ORF92). In this study, however, we could not clarify the localization of endogenous FIMP in *Izumo1* KO spermatozoa because anti-FIMP antibodies are not available for immunostaining and immunoblot analyses. Male infertility in *Fimp* KO mice may be caused by abnormalities in sperm protein or proteins other than IZUMO1. SPACA6 is a testis-specific transmembrane protein and was reported in a screening of male-infertile Tg mice that display sperm–oocyte fusion defects (52). However, no further phenotypic studies of *Spaca6* KO mice have been reported. Thus, a targeted mutagenesis experiment that produces *Spaca6* KO mice is required to reconfirm the reproductive phenotype and analyze whether it has a relationship to the *Fimp* KO phenotype. FIMP, IZUMO1, and SPACA6 are similar type I transmembrane proteins localized on the sperm head. FIMP and SPACA6 may also have an oocyte receptor counterpart, similar to IZUMO1. These proteins may function after the initiation of the IZUMO1–JUNO complex during sperm–oocyte fusion and/or as part of a larger IZUMO1-independent fusion-required complex. As mentioned previously, further studies are needed to examine the cause of male infertility in *Fimp* KO mice and to clarify the interaction of IZUMO1, SPACA6, and FIMP. Our findings support a potential role of human FIMP in sperm function and could be used to develop infertility treatments as well as male-specific contraceptives.

## Methods

### Animals.

All animal experiments were approved by the Animal Care and Use Committee of the Research Institute for Microbial Diseases, Osaka University. Human tissues were collected as nonhuman subject research by the Human Tissue Acquisition & Pathology Core at Baylor College of Medicine under the institutional review board-approved Protocol H-14435. Mice were maintained under a 12-h light/dark cycle (lights on from 8:00 to 20:00). Wild-type mice were purchased from CLEA Japan (Tokyo, Japan) and Japan SLC (Shizuoka, Japan). In this study, we generated genetically modified mouse lines, *Fimp* KO mice (B6D2-*4930451I11Rik*<em1Osb>); RBRC09958, FIMP TM-deleted mice (B6D2-*4930451I11Rik*<em2Osb>); RBRC10114, and *Fimp-mCherry* and *CAG/Acr-EGFP* double Tg mice (B6D2-Tg(*Clgn-4930451I11Rik/mCherry*)1OsbTg(*CAG/Acr-Egfp*)C3-N01-FJ002Osb); RBRC10120. These were deposited to the RIKEN BioResource Research Center (https://mus.brc.riken.jp/en/) and the Center for Animal Resources and Development, Kumamoto University (http://card.medic.kumamoto-u.ac.jp/card/english/).

### Generation of *4930451I11Rik* Mutant Mice with CRISPR/Cas9.

*4930451I11Rik* KO mice were produced by microinjection of the pX330 plasmid (https://www.addgene.org/42230/) into mouse embryos, as described previously (50, 51). A search for sgRNA and off-target sequences was performed using CRISPRdirect software (https://crispr.dbcls.jp/) (53). The sgRNA sequence used for microinjection were: 5′-GGA​ACC​CGG​CCC​TCG​GTT​TC-3′ for the second exon of *4930451I11Rik* (targeted for the common exon of two variants) and 5′-CCT​CAG​GAA​CTT​CCA​GAA​AG-3′ for the fourth exon of *4930451I11Rik*. Each sgRNA was injected into the pronuclei of fertilized eggs. The 2-cell stage embryos were transferred into the oviducts of pseudopregnant ICR females the next day. *4930451I11Rik* KO mice had a 1-bp deletion (5′-T-3′) in the second exon. 4930451I11Rik TM-deleted mice had a 246-bp deletion in the fourth exon. Both a 563-bp band as the wild-type allele and a 317-bp band as the TM-deleted allele were amplified by PCR. The primers used are listed in *SI Appendix*, Table S1. Detailed genotype information of mutant mouse lines is shown in the *SI Appendix*, Figs. S2*A* and S3*A*.

### Sperm–Oocyte Fusion Assay.

Sperm–oocyte fusion assay using mouse spermatozoa was performed as described previously (12). Briefly, ZP removal was performed by incubating the cumulus oocyte complex with collagenase (Sigma Aldrich, MO) at 100 μg/mL for 10 min. Hoechst 33342 preloaded ZP-free oocytes were inseminated with 2 × 10^5^ cells/mL capacitated spermatozoa for 30 min. After fixing with 0.25% glutaraldehyde and washing with TYH medium, the number of fused spermatozoa was counted under a fluorescence microscope.

### Generation of *Fimp-mCherry* Tg Rescue Mice.

To make the Tg rescue construct, the TM form of mouse *Fimp* cDNA was amplified by PCR, using wild-type testis cDNA, fused with the amplified *mCherry* cDNA at the C terminus, and introduced downstream of the mouse *Clgn* promoter in pClgn1.1 vector (54). The KpnI- and SacI-digested transgene fragment (1.9 kb) was purified by gel electrophoresis and microinjected into the pronuclei of fertilized eggs from *Fimp* heterozygous KO mice using standard methods to create the *Fimp-mCherry* Tg rescue mouse line [B6D2-Tg(*Clgn-4930451I11Rik/mCherry*)1Osb]. For genotyping, a 600-bp band corresponding to the Tg allele was amplified by PCR, using primers as listed in *SI Appendix*, Table S1.

### Statistical Analysis.

Statistical analyses were performed using Student’s *t* test inserted into Microsoft Excel after the data were tested for normality of distribution. Differences were considered significant at *P* < 0.01.

### Data Availability Statement.

All data are included in the paper or its supporting appendix.

## Supplementary Material

Supplementary File
