# Supplementary material for: Spermatozoa lacking Fertilization Influencing Membrane Protein (FIMP) fail to fuse with oocytes in mice
Source: Proc Natl Acad Sci U S A. 2020 Apr 15;117(17):9393–400. doi: 10.1073/pnas.1917060117 (PMC7196805; doi:10.1073/pnas.1917060117)
Supplement: Supplementary File [file pnas.1917060117.sapp.pdf]

PNAS
www.pnas.org

Supplementary Information for

Spermatozoa lacking Fertilization Influencing Membrane Protein (FIMP) fail to fuse with oocytes in mice

Yoshitaka Fujihara[1,2,3], Yonggang Lu[1], Taichi Noda[1], Asami Oji[1,4], Tamara Larasati[1,5], Kanako Kojima-Kita[1,5], Zhifeng Yu[2], Ryan M. Matzuk[2], Martin M. Matzuk[2]* and Masahito Ikawa[1,5,6]*

[1] Research Institute for Microbial Diseases, Osaka University, 3-1 Yamadaoka, Suita, Osaka 565-0871, Japan. [2] Center for Drug Discovery and Department of Pathology & Immunology, Baylor College of Medicine, One Baylor Plaza, Houston, TX 77030. [3] Department of Bioscience and Genetics, National Cerebral and Cardiovascular Center, 6-1 Kishibeshinmachi, Suita, Osaka 564-8565, Japan. [4] Laboratory for Developmental Epigenetics, RIKEN Center for Biosystems Dynamics Research, 2-2-3 Minatojima-minamimachi, Chuo-ku, Kobe, Hyogo 650-0047, Japan. [5] Graduate School of Medicine, Osaka University, 2-2 Yamadaoka, Suita, Osaka 565-0871, Japan. [6] The Institute of Medical Science, The University of Tokyo, 4-6-1 Shirokanedai, Minato-ku, Tokyo 108-8639, Japan.

* Corresponding authors:
Martin M. Matzuk, MD, PhD and Masahito Ikawa, PhD
Email: mmatzuk@bcm.edu and ikawa@biken.osaka-u.ac.jp

This PDF file includes:

Supplementary text
Figures S1 to S5
Tables S1
SI References

Supplementary Information Text

RT-PCR analysis. Mouse cDNA was prepared from multiple adult tissues of wild-type mice (1). Briefly, using TRIzol reagent (Invitrogen, CA, USA), total RNA was isolated from multiple adult tissues of wild-type mice and multiple adult human tissues obtained from the Human Tissue Acquisition & Pathology Core. Informed consent of these human tissues was obtained. Mouse and human cDNA were prepared using SuperScript III Reverse Transcriptase (Invitrogen) following the manufacturer's instruction. The amplification conditions were 1 min at 94℃, followed by 30-35 cycles of 94℃ for 30 sec, 65℃ for 30 sec, and 72℃ for 30 sec, with a final 7-min extension at 72℃. The primers used are listed in Table S1.

Amplification and sequencing of the mouse *Fimp* variants. The cDNAs encoding the mouse *Fimp* (*4930451I11Rik*) were amplified by PCR using wild-type mouse testis cDNAs as a template. Each amplified fragment (XbaI-XhoI) was introduced into a pBluescript II SK (+) vector and was performed by sequencing. The primers used are listed in Table S1.

Antibodies. The monoclonal antibodies used here were as described previously: KS64-125 for IZUMO1 (2). The BASIGIN (sc-9757) and FLAG (F1804) antibodies were purchased from Santa Cruz Biotechnology and Sigma Aldrich, respectively. Dilutions used were 1:200 to 1:300 for immunostaining and 1:500 to 1:1000 for immunoblot analysis.

Immunostaining. Immunostaining was performed as described previously (3, 4). A confocal microscopic observation was performed as described previously (5). Observation was used by BZ-X710 microscope (Keyence, Osaka, Japan) and Eclipse Ti microscope connected to a Nikon C2 confocal module (Nikon, Tokyo, Japan).

Immunoblot analysis. Immunoblot analysis was performed as described previously (6). Briefly, sperm samples were collected from cauda epididymis. These samples were homogenized in lysis buffer containing 1% Triton X-100 and 1% protease inhibitor (Nacalai Tesque, Kyoto, Japan) and then were centrifuged (10,000g for 20 min at 4℃), and the supernatants were collected. Protein lysates were separated by SDS/PAGE under reducing conditions and transferred to PVDF membranes (Merck Millipore, MA, USA). After blocking, blots were incubated with primary antibodies overnight at 4℃ and then incubated with secondary antibodies conjugated with horseradish-peroxidase. Detection was performed using Chemi-Lumi One Ultra (Nacalai Tesque).

Male fertility test. Sexually mature mutant male mice were caged with 2-month-old B6D2F1 or mutant females for several months, and the number of pups in each cage was counted within a week of birth. Average litter sizes (pups per plug) are presented as the number of total pups born divided by the number of plugs for each genotype.

Testis histology and sperm morphology. After breeding studies, males were killed by cervical dislocation following anesthesia. Testes were weighed individually. Testes were fixed in 4% paraformaldehyde in PBS and were processed for paraffin and OCT (Optimal Cutting Temperature) embeddings. Paraffin and frozen

sections were cut 5 µm. Paraffin sections stained with periodic acid-Schiff (PAS) and then counterstained with Mayer hematoxylin solution (Wako, Osaka, Japan). The cauda epididymal spermatozoa were dispersed in PBS, and subsequently sperm morphology was observed under a phase-contrast microscope (BX50, Olympus, Tokyo, Japan).

***In vitro* fertilization.** *In vitro* fertilization using mouse spermatozoa was performed as described previously (7).

HEK293T-oocyte binding assay. The open reading frame (ORF) of *Fimp* was cloned from mouse testis cDNA and conjugated with a Kozak sequence on the N-terminus and a FLAG tag on the C-terminus by RT-PCR. The PCR amplicon was inserted into the pCAG1.1 vector that contains a CAG promoter and a rabbit beta-globin polyadenylation signal. An expression vector bearing mCherry-tagged mouse *Izumo1* ORF was constructed in a similar manner. HEK293T cells were transfected with the expression vectors by calcium phosphate-DNA co-precipitation method. After 2 days of incubation, the HEK293T cells were resuspended in PBS containing 10 mM EDTA and stained with Hoechst33342. Oocytes were harvested from hormone-primed B6D2F1 females and treated with collagenase to remove zona pellucida (ZP). The ZP-free oocytes were incubated with 1×10^4 HEK293T cells in 100 µL TYH medium drops for 2 hours and observed under a Nikon Eclipse Ti confocal laser scanning microscope.

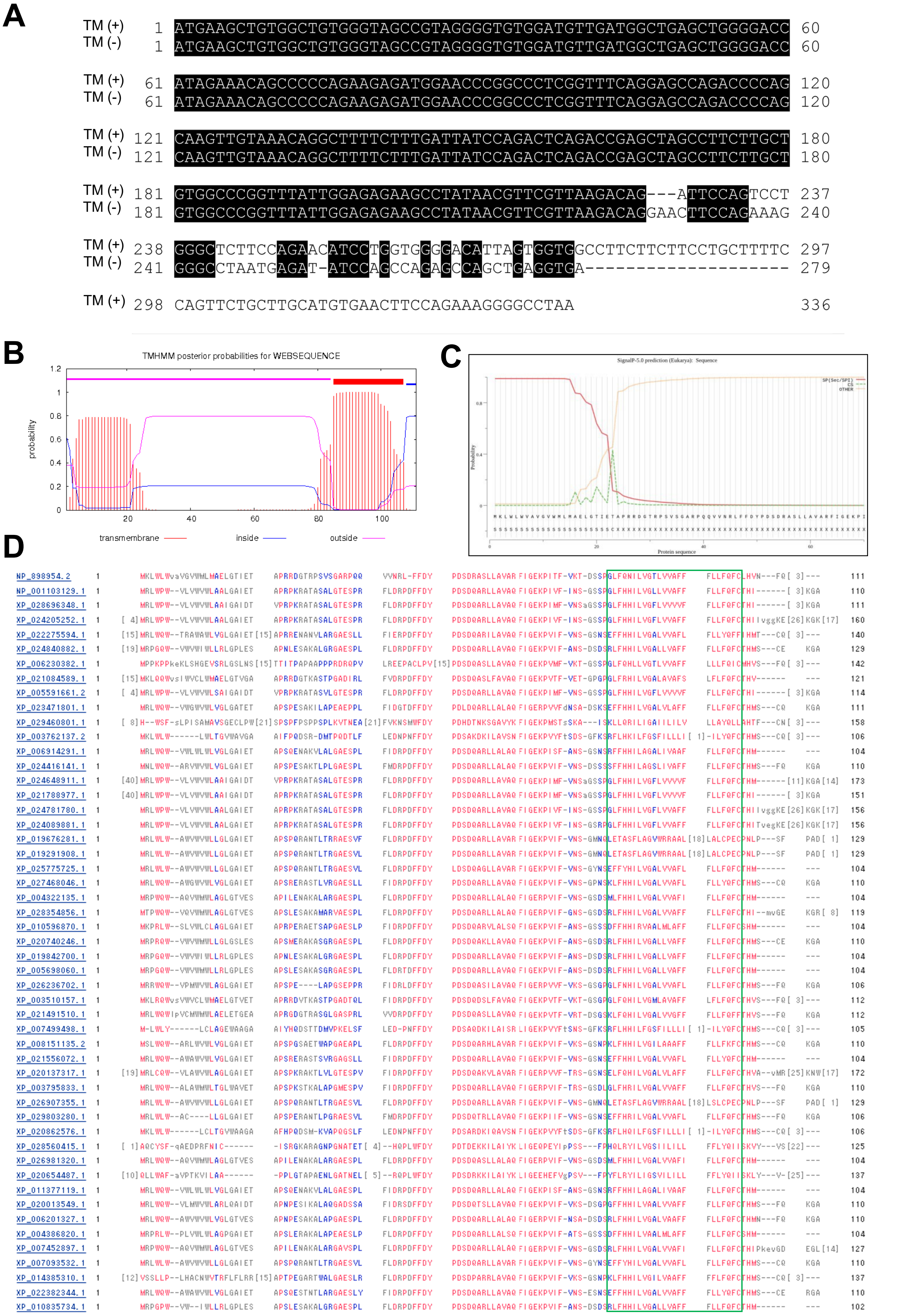
A
TM (+) 1 ATGAAGCTGTGGCTGTGGGTAGCCGTAGGGGTGTGGATGTTGATGGCTGAGCTGGGGACC 60
TM (-) 1 ATGAAGCTGTGGCTGTGGGTAGCCGTAGGGGTGTGGATGTTGATGGCTGAGCTGGGGACC 60
TM (+) 61 ATAGAAACAGCCCCCAGAAGAGATGGAACCCGGCCCTCGGTTTCAGGAGCCAGACCCCAG 120
TM (-) 61 ATAGAAACAGCCCCCAGAAGAGATGGAACCCGGCCCTCGGTTTCAGGAGCCAGACCCCAG 120
TM (+) 121 CAAGTTGTAAACAGGCTTTTCTTTGATTATCCAGACTCAGACCGAGCTAGCCTTCTTGCT 180
TM (-) 121 CAAGTTGTAAACAGGCTTTTCTTTGATTATCCAGACTCAGACCGAGCTAGCCTTCTTGCT 180
TM (+) 181 GTGGCCCGGTTTATTGGAGAGAAGCCTATAACGTTCGTTAAGACAG---ATTCCAGTCCT 237
TM (-) 181 GTGGCCCGGTTTATTGGAGAGAAGCCTATAACGTTCGTTAAGACAGGAACTTCCAGAAAG 240
TM (+) 238 GGGCTCTTCCAGAACATCCTGGTGGGGACATTAGTGGTGGCCTTCTTCTTCCTGCTTTTC 297
TM (-) 241 GGGCCTAATGAGAT-ATCCAGCCAGAGCCAGCTGAGGTGA-------------------- 279
TM (+) 298 CAGTTCTGCTTGCATGTGAACTTCCAGAAAGGGGCCTAA 336
B
TMHMM posterior probabilities for WEBSEQUENCE
probability
transmembrane
inside
outside
C
SignalP-5.0 prediction (Eukarya): Sequence
Probability
Protein sequence
SP(Sec/SPI)
CS
OTHER
D

Fig. S1. Nucleotide and amino acid sequence alignments of 4930451I11Rik. (A) Nucleotide sequence alignments of TM (+) and (-) forms of 4930451I11Rik. The sizes of the coding regions of TM (+) and (-) isoforms are 336 bp and 279 bp, respectively. Two variants match from the start codon (ATG) to the 226th nucleotide (75 of 111 amino acids in TM form). Black indicates a match in two sequences. (B, C) Protein structure prediction of the TM form of 4930451I11Rik. 4930451I11Rik is predicted to be a type I single-pass transmembrane protein with a signal peptide (1-23 aa), extracellular region (24-79 aa), transmembrane domain (80-102 aa), and intracellular region (103-111 aa). TMHMM-2.0 (B; https://services.healthtech.dtu.dk/service.php?TMHMM-2.0) and SignalP-5.0 (C; http://www.cbs.dtu.dk/services/SignalP-5.0/) software were used for this prediction. (D) Amino acid sequence similarity of 4930451I11Rik (C16ORF92) among 51 eutherian mammals [Mus musculus (TM form) (NP_898954.2), Homo sapiens (NP_001103129.1), Rattus norvegicus (XP_006230382.1), Macaca mulatta (XP_028696348.1), Pan troglodytes (XP_024205252.1), Pongo abelii (XP_024089881.1), Equus caballus (XP_023471801.1), Loxodonta africana (XP_010596870.1), Canis lupus familiaris (XP_022275594.1), Cricetulus griseus (XP_003510157.1), Sarcophilus harrisii (XP_003762137.2), Otolemur garnettii (XP_003795833.1), Pan paniscus (XP_024781780.1), Papio anubis (XP_021788977.1), Felis catus (XP_019676281.1), Tursiops truncates (XP_004322135.1), Trichechus manatus latirostris (XP_004386820.1), Mesocricetus auratus (XP_021084589.1), Bos taurus (XP_024840882.1), Macaca fascicularis (XP_005591661.2), Capra hircus (XP_005698060.1), Myotis brandtii (XP_014385310.1), Vicugna pacos (XP_006201327.1), Pteropus Alecto (XP_006914291.1), Panthera tigris altaica (XP_007093532.1), Lipotes vexillifer (XP_007452897.1), Monodelphis domestica (XP_007499498.1), Eptesicus fuscus (XP_008151135.2), Bison bison bison (XP_010835734.1), Pteropus vampyrus (XP_011377119.1), Macaca nemestrina (XP_024648911.1), Microcebus murinus (XP_020137317.1), Panthera pardus (XP_019291908.1), Bos indicus (XP_019842700.1), Castor canadensis (XP_020013549.1), Pogona vitticeps (XP_020654487.1), Odocoileus virgunianus texanus (XP_020740246.1), Phascolarctos cinereus (XP_020862576.1), Meriones unguiculatus (XP_021491510.1), Neomonachus schauinslandi (XP_021556072.1), Enhydra lutris kenyoni (XP_022382344.1), Desmodus rotundus (XP_024416141.1), Puma concolor (XP_025775725.1), Urocitellus parryii (XP_026236702.1), Acinonyx jubatus (XP_026907355.1), Lagenorhynchus obliquidens (XP_026981320.1), Zalophus californianus (XP_027468046.1), Physeter catodon (XP_028354856.1), Podarcis muralis (XP_028560415.1), Rhinatrema bivittatum (XP_029460801.1), Suricata suricatta (XP_029803280.1)]. The green box indicates a putative transmembrane domain. 4930451I11Rik is conserved broadly in mammals.

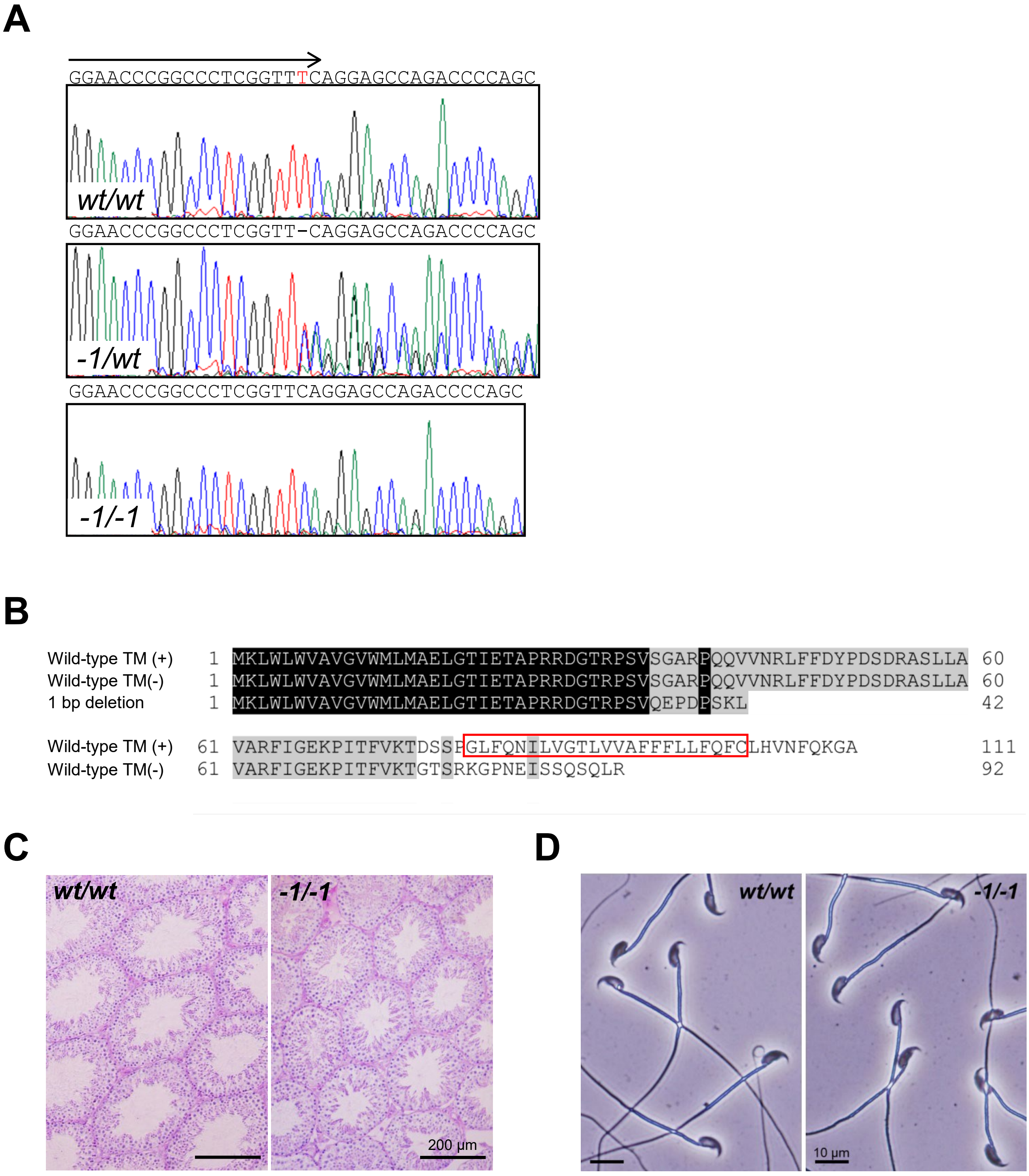
A
GGAACCCGGCCCTCGGTTTCAGGAGCCAGACCCCAGC
wt/wt
GGAACCCGGCCCTCGGTT-CAGGAGCCAGACCCCAGC
-1/wt
GGAACCCGGCCCTCGGTTCAGGAGCCAGACCCCAGC
-1/-1
B
Wild-type TM (+) 1 MKLWLWVAVGVWMLMAELGTIETAPRRDGTRPSVSGARPQQVVNRLFFDYPDSDRASLLA 60
Wild-type TM(-) 1 MKLWLWVAVGVWMLMAELGTIETAPRRDGTRPSVSGARPQQVVNRLFFDYPDSDRASLLA 60
1 bp deletion 1 MKLWLWVAVGVWMLMAELGTIETAPRRDGTRPSVQEPDPSKL 42
Wild-type TM (+) 61 VARFIGEKPITFVKTDSSPGLFQNILVGTLVVAFFFLLFQFCLHVNFQKGA 111
Wild-type TM(-) 61 VARFIGEKPITFVKTGTSRKGPNEISSQSQLR 92
C
wt/wt
-1/-1
200 µm
D
wt/wt
-1/-1
10 µm

Fig. S2. Generation of *4930451I11Rik* KO mice with CRISPR/Cas9. (A) Direct sequencing waveforms of the 1 bp deletion (5'-T-3', indicated by red letter) in the second exon of *4930451I11Rik* gene. Arrow indicates gRNA sequence. (B) Amino acid sequence of *4930451I11Rik* KO mice. The 1 bp deletion caused a frameshift mutation leading to a premature termination codon after the 42nd amino acid of both isoforms (111 amino acids in wild-type TM (+) form and 92 amino acids in wild-type TM (-) form). The putative transmembrane domain is indicated by the red box. Black indicates a match in all sequences, whereas gray indicates a match in two sequences. (C) Representative testicular histology sections stained with hematoxylin and eosin. Spermatogenesis in *4930451I11Rik* KO (-1/-1) mice is normal compared to that in wild-type (wt) mice. Scale bars: 200 μm. (D) Cauda epididymal spermatozoa from wild-type and *4930451I11Rik* KO mice. Sperm morphology in *4930451I11Rik* KO (-1/-1) mice is normal compared to that in wild-type (wt) mice. Scale bars: 10 μm.

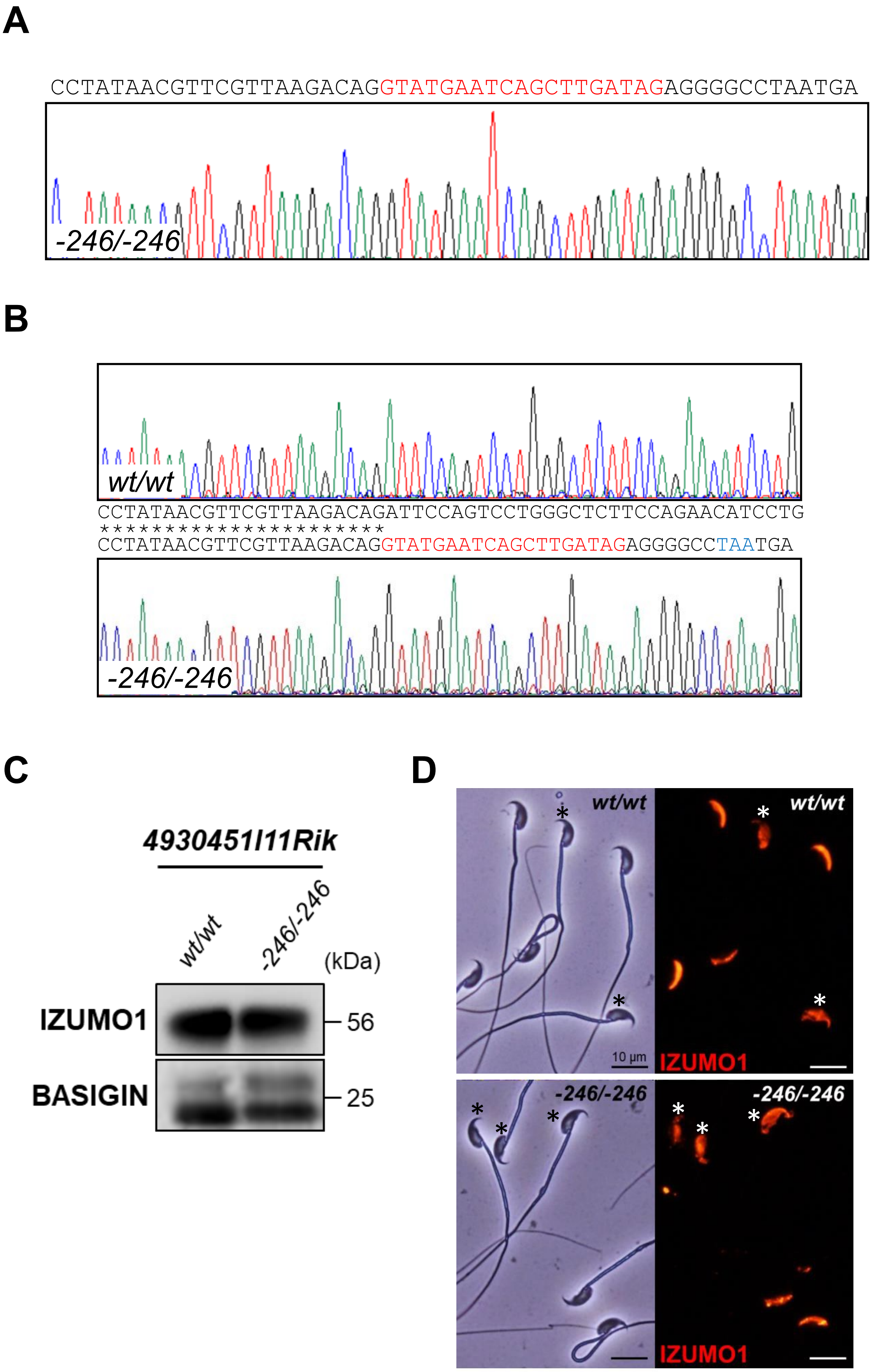
A
CCTATAACGTTCGTTAAGACAGGTATGAATCAGCTTGATAGAGGGGCCTAATGA
-246/-246
B
wt/wt
CCTATAACGTTCGTTAAGACAGATTCCAGTCCTGGGCTCTTCCAGAACATCCTG
****
CCTATAACGTTCGTTAAGACAGGTATGAATCAGCTTGATAGAGGGGCCTAATGA
-246/-246
C
4930451I11Rik
wt/wt
-246/-246
(kDa)
IZUMO1
56
BASIGIN
25
D
wt/wt
wt/wt
10 μm
IZUMO1
-246/-246
-246/-246
IZUMO1

Fig. S3. Generation of 4930451I11Rik TM-deleted mice with CRISPR/Cas9. (A) Direct sequencing waveforms of the 246 bp deletions around the third exon of the *4930451I11Rik* gene. Exons and introns are indicated by black and red letters, respectively. (B) Direct sequencing waveforms from testis cDNA in 4930451I11Rik TM-deleted mice. A part of the second intron (5'-GTATGAATCAGCTTGATAG-3') and stop codon are indicated by red and blue letters, respectively. 19 bps of the second intron were abnormally expressed in the testis of 4930451I11Rik TM-deleted mice. Nucleotides that match between wild-type and TM-deleted (-246/-246) mice are indicated by asterisks (226 of 336 bp match in wild-type mice). (C) Immunoblot analysis of IZUMO1 using sperm lysates from 4930451I11Rik TM-deleted mice. There are no differences of IZUMO1 localization between wild-type and 4930451I11Rik TM-deleted spermatozoa. BASIGIN was used as a loading control. (D) Immunostaining of IZUMO1 in 4930451I11Rik TM-deleted spermatozoa. IZUMO1 is used as a marker of the acrosome reaction. Acrosome reaction occurred in 4930451I11Rik TM-deleted (-246/-246) spermatozoa. Acrosome-reacted spermatozoa (determined by whole head pattern of IZUMO1) are indicated by asterisks. Scale bars: 10 μm.

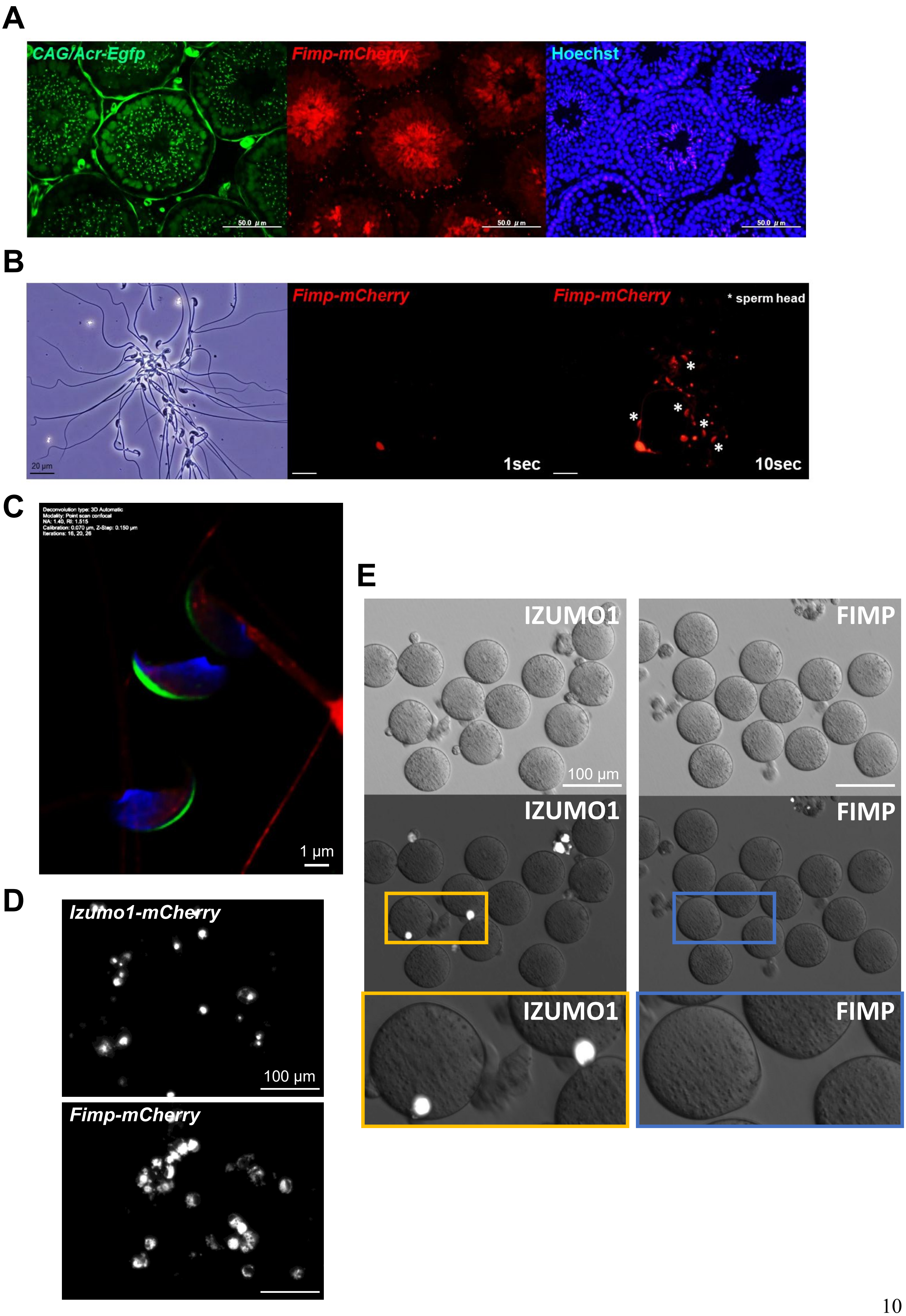
A
CAG/Acr-Egfp
Fimp-mCherry
Hoechst
50.0 µm
50.0 µm
50.0 µm
B
20 µm
Fimp-mCherry
1sec
Fimp-mCherry
* sperm head
10sec
C
Deconvolution type: 3D Automatic
Modality: Point scan confocal
NA: 1.40, RI: 1.515
Calibration: 0.070 µm, Z-Step: 0.150 µm
Iterations: 16, 20, 26
1 µm
D
Izumo1-mCherry
100 µm
Fimp-mCherry
E
IZUMO1
FIMP
100 µm
IZUMO1
FIMP
IZUMO1
FIMP

Fig. S4. Analysis of *Fimp-mCherry* transgenic mice and COS-7-oocyte binding assay. (A) Representative testicular histology of frozen sections of *Fimp-mCherry* transgenic (Tg) mice. The *Fimp-mCherry* Tg mice had another transgene [*CAG/Acr-Egfp*] which expressed EGFP throughout the entirety of the mouse and sperm acrosome. Spermatogenesis in *Fimp* KO (-1/-1) mice looked normal compared to that in wild-type mice. Scale bars: 50 µm. (B) Fluorescent observation of *Fimp-mCherry* Tg mouse spermatozoa. Although mCherry signals could not be detected on the sperm head using a short exposure time (1 sec), the signals could observe on sperm head using a longer duration of exposure (10 sec). Most of the FIMP-mCherry protein in cauda epididymal spermatozoa was concentrated in cytoplasmic droplets. Sperm heads with red signals are indicated by asterisks. Scale bars: 20 µm. (C) Confocal microscopic observation of *Fimp-mCherry* Tg mouse spermatozoa. The TM form of FIMP-mCherry fused protein is detected on the equatorial segment of the sperm head in acrosome-intact spermatozoa. Scale bar: 1 µm. (D) Observation of COS-7 cells expressing *Izumo1-mCherry* (upper panel, white signals) and *Fimp-mCherry* (lower panel, white signals) 2 days after transfection. Scale bars: 100 µm. (E) COS-7-oocyte binding assay. COS-7 cells expressing IZUMO1-mCherry (white signals) adhere to the plasma membrane of zona pellucida (ZP)-free oocytes, whereas the ones overexpressing FIMP-mCherry could not bind to the oocyte membrane. Scale bars: 100 µm. COS-7-oocyte binding assay was performed as described previously (8).

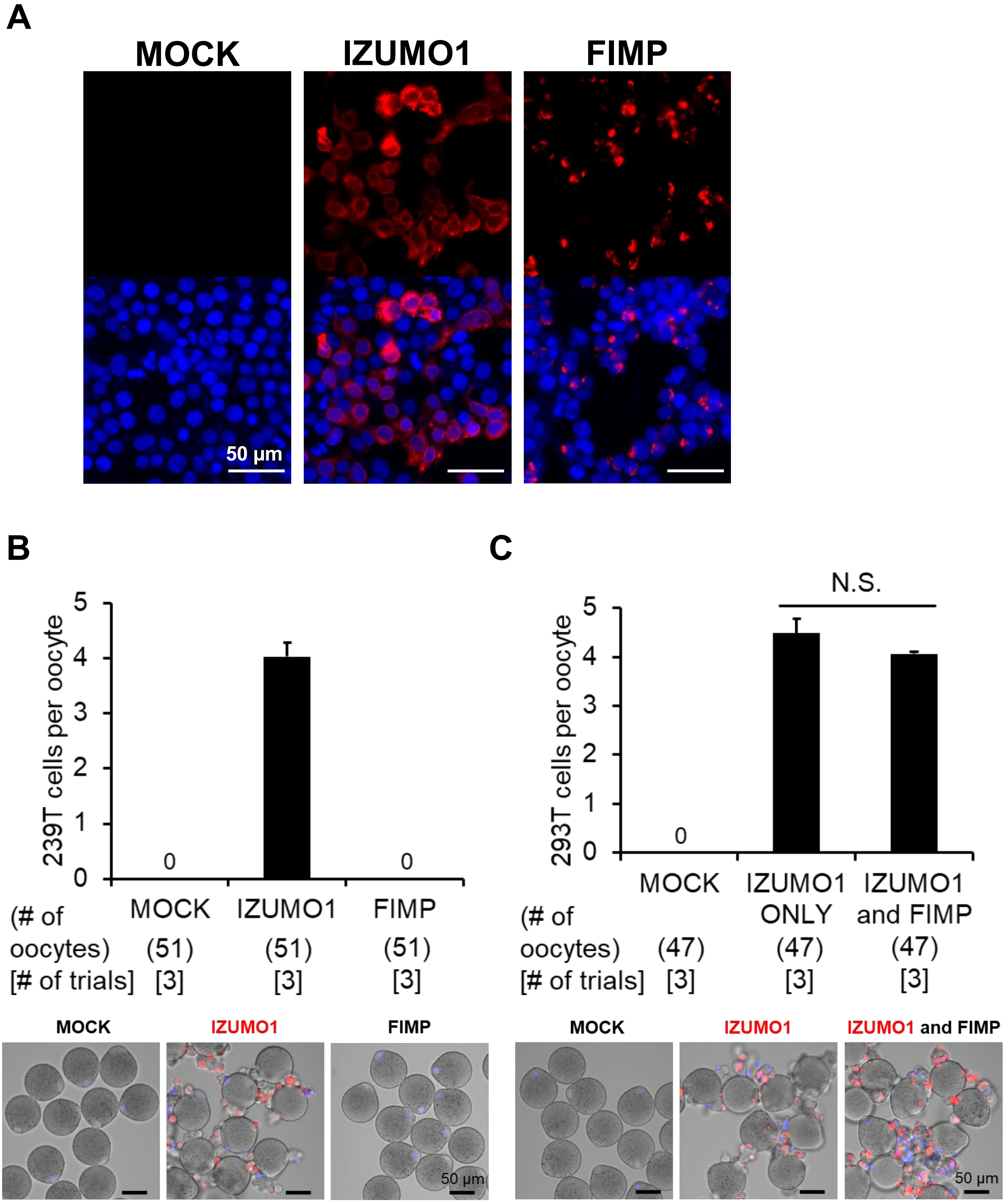
A
MOCK
IZUMO1
FIMP
50 μm
B
239T cells per oocyte
0
1
2
3
4
5
0
0
MOCK
IZUMO1
FIMP
(# of oocytes)
(51)
(51)
(51)
[# of trials]
[3]
[3]
[3]
C
N.S.
293T cells per oocyte
0
1
2
3
4
5
0
MOCK
IZUMO1 ONLY
IZUMO1 and FIMP
(# of oocytes)
(47)
(47)
(47)
[# of trials]
[3]
[3]
[3]
MOCK
IZUMO1
FIMP
50 μm
MOCK
IZUMO1
IZUMO1 and FIMP
50 μm

Fig. S5. HEK293T-oocyte binding assay. (A) Observation of HEK293T cells expressing *Izumo1-mCherry* (middle panel, red signals) and *Fimp-mCherry* (right panel, red signals) 2 days after transfection. Hoechst staining is indicated by blue signals. Scale bars: 50 μm. (B) HEK293T cells expressing mCherry-tagged IZUMO1 (red signals) adhere to the plasma membrane of ZP-free oocytes, whereas the ones overexpressing FLAG-tagged FIMP could not bind to the oocyte membrane. Hoechst staining is indicated by blue signals. Scale bars: 50 μm. (C) HEK293T cells solely expressing IZUMO1-mCherry showed a comparable index of oocyte binding compared with the ones simultaneously expressing IZUMO1-mCherry and FIMP-FLAG. Scale bars: 50 μm.

Table S1. List of primers.

Figure	Sequence (5' to 3')	Name
1A, 1B, 1D	TGTGGATGTTGATGGCTGAG	*4930451I11Rik*
	AGAAGGCAGGGTAGATGTGT	
	TGGATATGCCCTTGACTATAATGAG	*Hprt*
	TGGCAACATCAACAGGACTC	
1C	GATTATCCGGACTCAGACCAAG	Human *C16ORF92*
	CAGGATGTGATGGAAGAGCC	
	AATCCCATCACCATCTTCCAG	Human *GAPDH*
	ATGACCCTTTTGGCTCCC	
3A, 3B, S3A	GCCTTCTTGCTGTGGCCCGG	Primer #1
	CGCCTGCAGCCTGGGAGG	Primer #2
3C, S3B	ATGAAGCTGTGGCTGTGGGTAGC	TM-deletion
	TCACCTCAGCTGGCTCTGGCTG	
4A	AATCTAGAGCCGCCATGAAGCTGTGGCTGTGGGTAGC	TM form for Tg
	TTAAGCTTGGCCCCTTTCTGGAAGTTCACATGC	
	TTAAGCTTGTGAGCAAGGGCGAGGAGGATAAC	mCherry for Tg
	TTCTCGAGTTACTTGTACAGCTCGTCCATGCCGC	
4A, 4B	TTGAGCGGGCCGCTTGCGCACTGG	Primer #3
	GGCCCCTTTCTGGAAGTTCACATGC	Primer #4
S1A	ATGAAGCTGTGGCTGTGGGTAGC	*4930451I11Rik* (TM form)
	TTAGGCCCCTTTCTGGAAGTTCACATG	
	ATGAAGCTGTGGCTGTGGGTAGC	*4930451I11Rik* (secreted form)
	TCACCTCAGCTGGCTCTGGCTG	
S2A	GGCCCAGCCCCAGGCC	*4930451I11Rik* genotyping
	GGAGATCAGATGAGGAGGGACACATAGG	

References

1. Y. Fujihara *et al.*, Sperm equatorial segment protein 1, SPESP1, is required for fully fertile sperm in mouse. *J Cell Sci* **123**, 1531-1536 (2010).
2. Y. Fujihara *et al.*, Expression of TEX101, regulated by ACE, is essential for the production of fertile mouse spermatozoa. *Proc Natl Acad Sci U S A* **110**, 8111-8116 (2013).
3. M. Muto, Y. Fujihara, T. Tobita, D. Kiyozumi, M. Ikawa, Lentiviral Vector-Mediated Complementation Restored Fetal Viability but Not Placental Hyperplasia in Plac1-Deficient Mice. *Biol Reprod* **94**, 6 (2016).
4. Y. Fujihara *et al.*, SPACA1-deficient male mice are infertile with abnormally shaped sperm heads reminiscent of globozoospermia. *Development* **139**, 3583-3589 (2012).
5. Y. Satouh, N. Inoue, M. Ikawa, M. Okabe, Visualization of the moment of mouse sperm-egg fusion and dynamic localization of IZUMO1. *J Cell Sci* **125**, 4985-4990 (2012).
6. Y. Fujihara, A. Oji, T. Larasati, K. Kojima-Kita, M. Ikawa, Human Globozoospermia-Related Gene Spata16 Is Required for Sperm Formation Revealed by CRISPR/Cas9-Mediated Mouse Models. *Int J Mol Sci* **18** (2017).
7. K. Tokuhiro, M. Ikawa, A. M. Benham, M. Okabe, Protein disulfide isomerase homolog PDILT is required for quality control of sperm membrane protein ADAM3 and male fertility [corrected]. *Proc Natl Acad Sci U S A* **109**, 3850-3855 (2012).
8. N. Inoue *et al.*, Molecular dissection of IZUMO1, a sperm protein essential for sperm-egg fusion. *Development* **140**, 3221-3229 (2013).
